# The moderating effect of cognitive function on the relationship between depression and behavioral activation in older adults

**DOI:** 10.1002/alz70858_102497

**Published:** 2025-12-25

**Authors:** Yeonjoo Nam, YoungImm Choi, Sungmin Son, Mose Hwang, Taehui Kim

**Affiliations:** ^1^ Yonsei university, Wonju‐si, Gangwon‐do, Korea, Republic of (South); ^2^ Yonsei University Wonju Severance Christian Hospital, Wonju‐si, Gangwon‐do, Korea, Republic of (South); ^3^ Daeduk University, Daejeon, Daejeon, Korea, Republic of (South); ^4^ Chungnam National University, Daejeon, Daejeon, Korea, Republic of (South)

## Abstract

**Background:**

Depression in older adults is closely associated with cognitive decline, highlighting the importance of effective management. The behavioral activation model in older adults suggests a bidirectional relationship between functional decline, activity restriction, and common geriatric conditions. Behavioral activation disrupts this cycle by promoting engagement in valued activities, thereby reducing activity restriction and avoidance. This study examined the moderating effect of cognitive function in the relationship between depression and behavioral activation in community‐dwelling older adults in Korea.

**Method:**

Participants (*n* = 253) were enrolled from government‐sponsored senior welfare institutions due to depression, loneliness, or suicidal ideation. Behavioral activation was assessed using a short version of the Behavioral Activation for Depression Scale(BADS), validated for Korean older adults in a previous study. Additionally, the Valuing Questionnaire(VQ), Geriatric Depression Scale(GDS), and Cognitive Impairment Screening Test(CIST) were used. Standardized scores were used for statistical analysis. Correlation and multiple regression analyses examined the relationships between variables, with hierarchical regression used to test the moderating role of cognitive function.

**Result:**

The sample comprised 212 women (mean age=78.49, SD=5.26) and 41 men (mean age=75.10, SD=5.70). Of the participants, 87.7% were diagnosed with depression. Significant positive correlations were observed between the Avoidacne/Rumination(AR), Work impairment(WI), and Social impairment(SI) subfactors of BADS, with negative correlations to the Activation(AC) subfactor. The AR subfactor was negatively correlated with both the VQ and CIST, but positively with the GDS. The regression model explained 43.4% of the variance in depression. VQ had the strongest impact followed by the AC and SI subfactor. Cognitive function moderated the relationship between the AR, WI, SI subfactors and depression, with lower cognitive function amplifying effects of subfactors. The AR‐CIST interaction explained an additional 1.2% of depression variance, while the WI‐CIST and SI‐CIST interactions explained 1.6% and 2.9%, respectively. The AC‐CIST interaction was not significant.

**Conclusion:**

Higher cognitive function mitigates the depressive effects of rumination and impairments in work and social relationships. In contrast, lower cognitive function exacerbates these effects. Cognitive function did not moderate the relationship between behavior activation and depression. These findings suggest that interventions focused on increasing activity levels may effectively alleviate depression, regardless of cognitive function.

